# Acknowledgment of Reviewers

**DOI:** 10.2188/jea.JE20240409

**Published:** 2024-12-05

The following scientists assisted the journal by reviewing manuscripts during the period November 1, 2023 to October 31, 2024.

We gratefully acknowledge their critical evaluation and valuable assistance in selecting articles for publication.

**Table:** 

Abe, Sarah K.
Abe, Takumi
Aizawa, Yuta
Anzai, Tatsuhiko
Ariyada, Kenichi
Asakura, Keiko
Asayama, Kei
Bui, Chi Linh
Chiba, Yasutaka
Choi, Eunji
Davigo, Michele
Diemer, Elizabeth
Fujii, Hideki
Fujii, Ryosuke
Fujimori, Maiko
Fujita, Koji
Fukui, Keisuke
Fukushima, Wakaba
Gallus, Silvano
Goto, Atsushi
Gunathilake, Madhawa
Hachiya, Tsuyoshi
Hanyuda, Akiko
Harada, Kazuhiro
Harada, Sei
Hatanaka, Sho
Hayashi, Katsuma
Hikichi, Hiroyuki
Hirata, Takumi
Hirokawa, Kumi
Honda, Takanori
Hori, Megumi
Horita, Nobuyuki
Hosono, Satoyo
Hosozawa, Mariko
Hsieh, Cheng-Yang
Huang, Yubei
Ide, Kazushige
Igeta, Masataka
Iida, Miho
Ikeda, Nayu
Imai, Tomoko
Imaizumi, Takahiro
Imoto, Waki
Inoue, Kosuke
Inoue, Norihiko
Inoue, Yosuke
Ishii, Ryota
Ishimaru, Miho
Ishitsuka, Kazue
Isumi, Aya
Iwanaga, Mai
Jang, Won Mo
Kakizaki, Masako
Katanoda, Kota
Kataoka, Yuki
Kato, Tuguhiko
Kawaguchi, Takumi
Kawahara, Takuya
Kidokoro, Tetsuhiro
Kihara, Tomomi
Kikuchi, Hiroyuki
Kim, Kyeezu
Kishimoto, Yoshimi
Kiuchi, Sakura
Kiyohara, Kosuke
Koda, Masahide
Komatsu, Masayo
Komiyama, Jun
Komukai, Sho
Kondo, Takaaki
Koriyama, Chihaya
Koyanagi, Yuriko
Kurokawa, Naoyuki
Kuwabara, Masanari
Kyan, Akira
Li, Jiaqi
Liao, Li-Na
Lin, Yingsong
Ma, Enbo
Maeda, Eri
Matsumoto, Naomi
Matsushita, Munehiro
Matsuyama, Yusuke
Matsuzaka, Masashi
Matt, Georg E
Mezawa, Hidetoshi
Mischlinger, Johannes
Miyawaki, Atsushi
Mohta, Alpana
Momma, Haruki
Mori, Nagisa
Morisaki, Naho
Murai, Utako
Murakami, Keiko
Nagayoshi, Mako
Nakagomi, Atsushi
Nakamoto, Isuzu
Nakamoto, Mariko
Nakamura, Koshi
Nakano, Takashi
Nakao, Yoko
Nakashima, Kei
Nakatani, Eiji
Narita, Zui
Nemoto, Yuta
Nishijima, Takeshi
Oba, Shino
Ochi, Manami
Oh, Chang-Mo
Ohbe, Hiroyuki
Ohfuji, Satoko
Okada, Yohei
Okubo, Ryo
Okumura, Yasuyuki
Omori, Hisamitsu
Oono, Fumi
Osawa, Masaki
Otsuka, Rei
Otsuka, Yuichiro
Possenti, Irene
Saito, Eiko
Saito, Isao
Sata, Mizuki
Sato, Haruka
Sato, Kyoko
Sato, Shuntaro
Scala, Marco
Shin, Jung-ho
Shinozaki, Tomohiro
Shobugawa, Yugo
Song, Zean
Stival, Chiara
Sugawara, Yuka
Sun, Yu
Suzuki, Hiroyuki
Suzuki, Takayuki
Suzuki, Yuka
Tabuchi, Takahiro
Taira, Kento
Takeuchi, Jiro
Tanabe, Naoya
Tanaka, Hirokazu
Tanaka, Junko
Tanaka, Kotone
Tang, Julian
Taniguchi, Yuta
Taniyama, Yukari
Terui, Toshihiro
Tian, Haodong
Tsuboya, Toru
Tsuge, Hiroshi
Tsuji, Taishi
Ugai, Satoko
Ukawa, Shigekazu
Umesawa, Mitsumasa
Uno, Shunsuke
Uozumi, Ryuji
Uraguchi, Kensuke
Utada, Mai
Utsunomiya, Takeshi
Wakai, Kenji
Waki, Takashi
Wakui, Tomoko
Watai, Izumi
Wu, Lijun
Xu, Wanghong
Yachi, Sen
Yamada, Takuya
Yamaji, Taiki
Yamakawa, Michiyo
Yamamoto, Takafumi
Yatsuya, Hiroshi
Ye, Yuanzi
Yon, Dong Keon
Yoshida, Kazuhiro
Yoshizaki, Takahiro

